# Bridging storytelling traditions with digital technology

**DOI:** 10.3402/ijch.v72i0.20717

**Published:** 2013-08-05

**Authors:** Melany Cueva, Regina Kuhnley, Laura J. Revels, Katie Cueva, Mark Dignan, Anne P. Lanier

**Affiliations:** 1 Alaska Native Tribal Health Consortium, Anchorage, AK, USA; 2 Institute of Social and Economic Research, University of Alaska, Anchorage, AK, USA; 3 Markey Cancer Center, University of Kentucky, Lexington, KY, USA

**Keywords:** digital storytelling, Alaska Native, cancer education, Community Health Workers, health communications

## Abstract

**Objective:**

The purpose of this project was to learn how Community Health Workers (CHWs) in Alaska perceived digital storytelling as a component of the “Path to Understanding Cancer” curriculum and as a culturally respectful tool for sharing cancer-related health messages.

**Design:**

A pre-course written application, end-of-course written evaluation, and internet survey informed this project.

**Methods:**

Digital storytelling was included in seven 5-day cancer education courses (May 2009–2012) in which 67 CHWs each created a personal 2–3 minute cancer-related digital story. Participant-chosen digital story topics included tobacco cessation, the importance of recommended cancer screening exams, cancer survivorship, loss, grief and end-of-life comfort care, and self-care as patient care providers. All participants completed an end-of-course written evaluation. In July 2012, contact information was available for 48 participants, of whom 24 completed an internet survey.

**Results:**

All 67 participants successfully completed a digital story which they shared and discussed with course members. On the written post-course evaluation, all participants reported that combining digital storytelling with cancer education supported their learning and was a culturally respectful way to provide health messages. Additionally, 62 of 67 CHWs reported that the course increased their confidence to share cancer information with their communities. Up to 3 years post-course, all 24 CHW survey respondents reported they had shown their digital story. Of note, 23 of 24 CHWs also reported change in their own behavior as a result of the experience.

**Conclusions:**

All CHWs, regardless of computer skills, successfully created a digital story as part of the cancer education course. CHWs reported that digital stories enhanced their learning and were a culturally respectful way to share cancer-related information. Digital storytelling gave the power of the media into the hands of CHWs to increase their cancer knowledge, facilitate patient and community cancer conversations, and promote cancer awareness and wellness.

Digital stories come from our community – the voices and faces of our own people so it's more powerful, has more of an impact. It touches people's hearts. – Community Health Worker cancer education course participant.

Alaska Native people have a rich heritage of sharing knowledge and wisdom through stories. Stories have been used for generations to pass on traditions, life lessons and cultural values. Community Health Workers (CHWs) in Alaska have identified that stories create pathways for connecting people, facilitating knowledge and understanding, enhancing remembering, engendering creativity, expanding perspectives, envisioning the future and inspiring possibilities (1).

Digital storytelling combines oral storytelling traditions with computer technology. Since its inception in the early 1990s, digital storytelling has gained momentum as an education social advocacy tool (2). Paulo Freire, a Brazilian education theorist and social advocate, emphasized the importance of including learners’ thought and speech as the basis for developing critical understanding of personal experience, unequal conditions in society, and knowledge (3). Based on Freire's theoretical framework of empowerment (4), digital storytelling combines a person's recorded voice with their choice of pictures and music to bring the power of the media into the voices and hands of community members (5) as a meaningful and culturally relevant health messaging tool.

Alaska is the largest state in the US, comprising one-fifth of the landmass of the contiguous 48 states. Alaska Native and American Indian people represent approximately 15% of the state population, with approximately 60% of Alaska Native people living in rural communities (6). There are 178 communities separated from regional hospitals by vast stretches of tundra, water, glaciers, and mountains. Geographic remoteness significantly affects the ability of many Alaska Native people to access the full spectrum of cancer care: education, prevention services, early detection, diagnosis, treatment, support services, palliative, and end-of-life care. Because Alaska rural communities are small, ranging in size from 20 to 1,200 people, even a single cancer diagnosis can have a huge impact on a community.

Cancer was considered a rare disease among Alaska Native people as recently as the 1950s, but became the leading cause of mortality in the 1990s and remains so today (7). Contributing to cancer prevalence are modifiable risk factors experienced by many Alaska Native adults. Based on Alaska Behavioral Risk Factor Surveillance System data, as of 2009 37% of Alaska Native adults were current smokers, 26% reported no physical activity during the past month, 73% reported being overweight or obese, and 86% reported consuming less than 5 servings of fruits and vegetables per day (8).

Alaska has a unique network of village-based health care providers. CHWs, including approximately 600 Community Health Aides/Practitioners (CHA/Ps) (9) and 120 Behavioral Health Aides (BHAs), are community members chosen by their tribes to provide community health care. CHA/Ps and BHAs have requested cancer information to supplement their basic medical training. Equipped with culturally appropriate cancer education and resources, they are in an ideal position to support community members to modify behavioral risk factors, and advocate and refer individuals for recommended screening exams.

The purpose of this project was to learn how CHWs in Alaska perceived digital storytelling as a component of a cancer education curriculum and as a culturally respectful tool for sharing cancer-related health messages.

## Methods

An on-going dialogue with “Path to Understanding Cancer” course participants through a pre-course written application, end-of-course written evaluation, and internet survey informed this project. Cancer education participants were emailed throughout this dynamic process to celebrate their contributions and to learn their experience with combining digital storytelling and cancer education.

### “Path to Understanding Cancer” course description

The 5-day cancer education course was developed with and for Alaska's CHWs in 1999 to provide basic cancer information and is continually updated to include medically- accurate information and to respond to CHWs’ expressed needs (10). In 2009, the cancer education course was expanded to include digital storytelling which combines computer-based technology with storytelling. Stories are told using both oral and visual dimensions. Participants synthesize and integrate their cancer understandings with a personal narrative to create their own culturally relevant cancer health message. Cancer education course curriculum includes facts about cancer among Alaska Native people, self-care, healthy lifestyle choices to decrease cancer risk or prevent cancer, recommended screening exams to prevent cancer or find changes early that may be cancer, cancer diagnosis and treatment, pain assessment and management, and loss, grief and end-of-life comfort care. The course manual, *Understanding Cancer*, written in collaboration with medical providers in Alaska and CHWs, includes 9 sections: (a) Self-care; (b) Wellness ways to prevent and decrease cancer risk; (c) Cancer and our genes; (d) Understanding cancer basics; (e) Cancer treatments: what to expect; (f) Cancer pain: assessment and management; (g) Loss, grief, and end-of-life comfort care; (h) Resources and (i) Community activities (11).

Course faculty included a Registered Nurse with over 14 years of experience working in cancer education with Alaska Native people and an Alaska Native cancer survivor with digital storytelling expertise. Guest instructors, including a genetics counsellor and an oncology nurse, provided supplementary curriculum content. Additionally, tours of mammography and colorectal screening clinics were conducted.

### Participants

Courses were advertised through existing CHW networks which included state-wide list serves, newsletters, previous course participants, and CHW program leadership. Ten CHWs were selected for each course based upon the support of their regional health corporation, their interest in learning about cancer, and their ability to attend the entire course. As part of the pre-course written application, CHWs described the ways cancer had affected their lives and how they hoped to apply their knowledge as a result of course participation. As expressed by course participants on the pre-course application:

I think some people shy away from people affected by cancer, being they are not sure how to act around them. I for one would like to be there for the people and their family affected by this disease.

Being as busy as we are in the clinic, we don't do much on patient education regarding prevention of illness; we mostly focus on patient education on how to treat illness. I think this will help encourage me to talk to our patients more on prevention.

### End-of-course written evaluation

Upon completion of the cancer education course, participants were asked to complete a 3-page, written end-of-course evaluation, which included check-box and open-ended questions, to share their experiences with combining digital storytelling and cancer education. Questions specific to digital storytelling were added to the evaluation tool that had been successfully used in the course evaluations previously conducted by the project team, prior to inclusion of digital storytelling.

### Internet survey methods

To learn CHWs’ experience of digital storytelling over time, an internet survey was developed and implemented. The digital storytelling/cancer education course instructors developed the survey, with input from evaluation experts Regina Kuhnley and Dr. Mark Dignan. Questions were piloted with people available at the Anchorage Community Health Aide Program training centre, including CHAP instructors, CHA/Ps not involved with the course, and administrative support staff. The pilot survey reviewers checked for question readability and recommendations about the question wording to elicit the information we wanted to learn. The 10-question survey included check-box and open-ended questions.

Contact information was available for 48 of 67 course participants at the time of internet follow-up, which ranged from 3 months to 3 years post-course participation. Participants for the post-course internet survey were recruited via an email introduction that included a link to survey monkey and an attached paper survey. Three email reminders were sent over the course of 1 month to prompt survey completion. Two gift certificates of $50 each were given as a thank you to 2 randomly selected participants for taking the time to provide their ideas by completing the survey. Names were drawn from the list of respondents who chose to provide their name and contact information to be entered into the drawing. No data were linked to participants’ names or contact information. All responses are reported anonymously.

## Results

Between May 2009 and May 2012, seven 5-day cancer education courses were provided for 67 CHWs (62 women and 5 men) from throughout Alaska. The majority of Alaska CHWs are women. Participants included 38 Alaska Native people, including Athabascan, Aleut, Tlingit, Yupik, and Inupiaq Alaska Natives, as well as 8 American Indian people, including Sioux, Cherokee, Blackfeet, and Pawnee individuals. Thirteen participants were Caucasian, 2 Asian, and 1 Hispanic. Ages of participants ranged as follows: 10 (19–29 years old), 14 (30–39), 15 (40–49), 15 (50–59), and 12 (60 or older).

As part of cancer education course participation, all 67 CHWs were successful in creating a cancer-related digital story regardless of prior computer experience. Participants chose to tell personal stories with cancer messages related to tobacco cessation, the importance of recommended cancer screening exams, cancer survivorship, loss, grief and end-of-life comfort care, and self-care as patient care providers. Additionally, participants described cultural perspectives about cancer, reflecting traditional values, languages, traditions, ways of knowing, intergenerational knowledge transmission, and other cultural attributes. Through sharing their story, participants hoped that it would encourage other people to tell their story as a way to end the silence that often surrounds the challenging topic of cancer.

After completing their digital story, each participant responded in writing to the following statements: “I told this story because …” and “After watching my story, something I hope you think about …” In the words of a CHW:

I've seen too many people die of cancer. I believe in early screening. I know it won't detect all cancers or prevent all cancer deaths but it will decrease some. After watching my story, I hope people think about being screened. Think about the people that love you, who will be the one caring for you. Cancer affects everybody not just the person who has it.

### End-of-course written evaluations

All 67 course participants completed an anonymous end-of-course written evaluation. All participants recommended the cancer education course and affirmed ways that combining digital storytelling with cancer education worked for them, supported their learning and was culturally respectful. By creating a personal digital story, participants integrated their cancer knowledge with their personal experience to critically reflect upon the health message they wanted to share within their network of relationships. Consequently, participants described how story creation increased their cancer knowledge by requiring them to evaluate, synthesize, and apply their cancer knowledge into a digital story. Course participants wrote how they came to the course not knowing much about cancer and left the week knowing lots of information and with a tool to provide cancer information back home. CHWs wrote detailed information, describing the ways digital storytelling supported their learning which included the following:

By telling our own story it encouraged us to think about the subject, helped us to learn and speak out more.

My retention of information was greater than normal.

I know how to talk about cancer a lot easier. It makes it easier when you know the facts and how important it is to get screened and keep up with them. I'll be checking on the people back home.

CHW course participants enthusiastically expressed how combining digital storytelling with cancer education was culturally respectful. Storytelling was described as a way to share information that encouraged openness, tolerance, and sensitivity. Participants related how storytelling made it easier to hear the message by touching people's hearts.

As a result of cancer education course participation, 84% of participants (56 of 67) described ways they felt differently about cancer. Five people reported not feeling differently, 1 person was unsure and wrote that “the pain is still there but maybe lessens it some”, 2 responses were not specific to the question and 3 evaluations were blank. Some of the views expressed were as follows:

Less fear of the unknown. It's not as scary.

It's okay to say ‘Cancer’. Can talk about it now and say the word cancer. Feel more comfortable talking and learning.

It's okay to cry – okay to laugh. I can be more open. It is alright to grieve, share, and let go. It felt good to get that heavy thing I've been carrying out. It was a way for me to share my grief.

It's [Cancer] not a death sentence; it's not something we should hide from.

In response to the question, “By creating your own digital story do you feel more confident to share cancer education with people in your community?”, overall 93% (62 of 67) responded affirmatively. Representative CHW quotes are:

I have more information to share plus I have the courage – how to be there for someone and their family with cancer. More aware of survivors’ and caregivers’ feelings.

I learned a lot and will be much more aware of family, friends, and self-getting screenings and pay attention to their results. Also will offer to go with others.

I am the type not to speak in front of a crowd, so doing this helps a lot. Now I know what to say. I have a better knowledge of cancer. I have a tool to introduce the topic.

In response to the open-ended question, “Will you do anything differently as a result of cancer education for you, your family, and in your work?”, participants wrote detailed information describing intent to support healthy behaviors for themselves, their families and patients. Very few evaluations were left blank. 85% of participants (57 of 67) identified wellness changes they planned to make as a result of course participation for themselves, including having recommended screening exams (16 participants), eating healthier (10 participants) and being more physically active (7 participants). Additionally, 81% of participants (54 of 67) described ways they planned to support family health, including encouraging family screening activities (20 participants) and healthier eating (5 participants). As reported by 85% of CHW participants (57 of 67), the course strengthened their patient care practices, which included talking about cancer-related risk reduction behaviors and encouraging their patients to have recommended screening exams. They reported feeling more confident in their cancer knowledge and communication skills as well as being empowered to provide cancer education and support community wellness to decrease cancer risk. In the words of CHWs:

I learned to explain cancer to patients instead of the clinical gobbledygook, where you lose them in 2 minutes.

Speak out. Make sure to help people get their screening. Encourage screening.

### Internet survey results

Out of 67 course participants from the cancer education/digital storytelling courses offered May 2009–May 2012, 19 people were lost to follow-up, including 1 person who died from brain cancer. Of the remaining 48 course participants, 24 people completed the survey: 19 via survey monkey and 5 via emailed paper survey. Survey respondents included 22 females and 18 individuals who self-identified as Alaska Native.

Up to 3 years post-course, all 24 CHW survey respondents reported that they had shown their digital story to a variety of individuals. CHWs related they had shown their stories as part of community presentations, family gatherings, health fairs, school presentations, and clinic visits. Viewers included youth, elders, patients, community members, family, friends, co-workers, and tribal councils. Additionally, people had posted their stories on YouTube, Facebook pages, and other web sites. More recently, CHWs have posted their stories on the cancer education resource page of the akchap.org website. All survey respondents described digital storytelling as being a culturally respectful and effective health messaging tool as reflected in the following quote.

I think it is a beautiful way to get cancer health messages out to people because they can hear your voice, see your photos, and words. This is different than reading an article in the paper or hearing an ad on the radio. It's not some abstract or unknown entity sharing information – it's real people, people like you and me, maybe people that you know personally. It gives a face to the message and not just empty words.

Furthermore, 23 of 24 CHWs reported personal behavior change as a result of the experience, including having recommended screening exams (10 people), quitting tobacco (1 person), decreasing tobacco use (1 person), increasing physical activity (10 people), and eating healthier (12 people). In the words of a CHW:

The whole experience of cancer education and digital storytelling was very uplifting and it made me more aware of how cancer affected me in my decisions for myself and how I want to convey my message to my family about how they feel about it. I was given a tool in order to reach out to my closest family in a way that I wasn't able to before. I tried to talk about screening a few times but this is a way to open the topic without being confrontational. I'm showing them why I want this for them because I love them and care about their health. Digital storytelling is a very powerful medium for getting these messages across the barriers of other forms of communication.

## Discussion

The creation of digital stories was an integral part of a 5-day cancer education course for CHWs in Alaska. Digital storytelling weaves past storytelling traditions with new technologies, carrying people's stories across time – past, present and future. CHWs took their digital stories home as tools to share their heartfelt cancer-related health messages with people in their communities. These stories connect knowledge with emotion, linking realms of wisdom. As Wilson (12) advocates in *Research Is Ceremony*, indigenous methodologies ask that theses realms of wisdom be connected:

the western tradition teaches us to separate our head from our heart and our spirit. Therefore those roads and those lines of communications aren't linked up as they should be, and our cultures and our traditions teach us to hook those lines of communication up.

Stories can be understood as both a reflection of culture and the creation of behavior change (13). Course participants’ construction of digital stories influenced their post-course behavior, with self-reported changes that improved participants’ health, nurtured healthy activities for their families, and strengthened the ways they provided patient care. Paulo Freire discussed and implemented a model of empowerment in which people told and retold personal stories to transform behaviors (4). In a similar fashion, participants told and retold their own stories through the creation and discussion of digital stories, culminating in a final product that prompted many to alter their behavior to reflect healthier choices. The transformation of personal experiences into the physical reality of a digital story also allowed participants to objectively view their own experiences, opening a pathway for group discussions, course material and personal reflections to create new meaning from past experiences (13, 14). Participants expressed feelings of healing and renewal as a result of developing, discussing and showing their stories. The creation of digital stories leading to new reflections and behaviors highlights the use of digital storytelling as a powerful health messaging tool. Digital storytelling bridges historical cultural traditions of storytelling with technology to create new traditions for passing on knowledge to future generations. In the words of course participants:

We live in such a technology based world that digital storytelling naturally fits as a way to effectively tell our story.

Digital stories have a personal perspective that people understand. Our stories come from the heart. Community members lend their natural storytelling abilities to help educate others.

## Conclusions

Evaluation data from this project revealed that combining digital storytelling with cancer education for CHWs in Alaska feasibly enhanced learning in a culturally respectful way. Digital stories offered a forum for individual narratives to be expressed in both oral and visual dimensions, sharing the experience and culture of each participant. Participants in the cancer education courses expressed enthusiasm over learning how to use computer-based technology to create and tell a personal story, emphasizing the accessibility of digital storytelling to even those with minimal technological experience. Creating and telling of personal stories by course participants enhanced understanding of cancer, confidence with discussing cancer, and provided a tool to assist CHWs to share cancer-related health messages in their clinics and communities.

This project plants the seeds for future cultivation and exploration of the transformative power of digital storytelling. Qualitative methods will yield a deeper understanding of CHWs’ experience of creating a personal cancer-related digital story and how that influences health choices. Additionally, it is important to gain insight into how story creators continue to disseminate their digital stories and share them within their networks and communities. Much is to be learned about how digital story viewing affects cancer perceptions and health behaviors. Also yet to be fully explored are the elements of digital stories that make them a powerful tool for health messaging. Digital storytelling holds promise as an innovative and culturally respectful tool for health communication.
